# Investigating HLA haplotypes as a potential risk factor for nodding syndrome: A case-control study in the Mahenge area, Tanzania

**DOI:** 10.1371/journal.pntd.0012971

**Published:** 2025-11-26

**Authors:** Amber Hadermann, Dan Bhwana, Athanas D. Mhina, Luís-Jorge Amaral, Joseph N. Siewe Fodjo, Marie-Paule Emonds, Sarah Weckhuysen, Bruno P. Mmbando, Robert Colebunders

**Affiliations:** 1 Global Health Institute, University of Antwerp, Antwerp, Belgium; 2 National Institute for Medical Research, Tanga Research, Tanga, Tanzania; 3 Histocompatibility and Immunogenetics Laboratory, Belgian Red Cross-Flanders, Mechelen, Belgium; 4 Department of Neurology, University Hospital Antwerp, Belgium; 5 VIB-Center for Molecular Neurology, VIB, University of Antwerp, Belgium; 6 Translational Neurosciences, Faculty of Medicine and Health Science, University of Antwerp, Antwerp, Belgium; Institute of Cytology and Genetics SB RAS: FIC Institut citologii i genetiki Sibirskogo otdelenia Rossijskoj akademii nauk, RUSSIAN FEDERATION

## Abstract

**Background:**

Nodding syndrome (NS) is a disabling childhood-onset epilepsy occurring in onchocerciasis-endemic regions. High *Onchocerca volvulus* microfilarial loads in childhood are a key risk factor, but not all heavily infected individuals develop NS, suggesting a possible role for host genetic susceptibility. Human leukocyte antigen (HLA) haplotypes have been implicated in susceptibility to various infectious and autoimmune diseases, including onchocerciasis. We investigated potential associations between HLA haplotypes and onchocerciasis-associated epilepsy (OAE), including NS, in Tanzania.

**Methods:**

A case-control study was conducted in an onchocerciasis-endemic area in the Mahenge area, Tanzania, including 98 persons with epilepsy and 112 controls. DNA was extracted from dry blood spots and HLA sequence-based typing was performed by Histogenetics (Ossining, USA). A total of 11 HLA loci (HLA-A, -B, -C, -DRB1, -DRB3, -DRB4, -DRB5, -DQA1, -DQB1, -DPA1, and -DPB1) were exon sequenced. The HLA-typed dataset was analysed using pyHLA, including Bonferroni as multi-test adjustment, to test for associations with *O. volvulus* anti-Ov16 seropositivity, epilepsy, OAE and NS.

**Results:**

Anti-Ov16 seropositivity was significantly higher in cases than controls (76.5% vs 58.9%; p = 0.01). No HLA alleles were significantly associated with epilepsy, OAE, NS, or anti-Ov16 seropositivity after correction. Before adjustment, HLA-C07:01 appeared to be a risk factor for epilepsy, HLA-DQB106:02 was associated with OAE, HLA-B35:01 with NS, and HLA-C08:02 and DRB1*03:01 with anti-Ov16 seropositivity. Post-hoc power analysis indicated that substantially larger sample sizes would be required to confirm these associations.

**Conclusions:**

This study did not identify statistically significant HLA associations with epilepsy, OAE, NS, or *O. volvulus* exposure. However, several alleles—particularly HLA-B*35:01, also reported in a previous South Sudanese study—emerged as potential candidates for further investigation. Larger, multi-country studies with sufficient power are needed to clarify whether host genetic factors contribute to susceptibility to OAE and NS. Strengthening onchocerciasis elimination programmes remains essential, as NS is a preventable disease.

## Authors summary

Nodding syndrome (NS) is a devastating childhood-onset epilepsy found in areas with ongoing or past Onchocerca volvulus transmission. While heavy infection with the parasite is a clear risk factor, not everyone exposed develops NS, raising the question of whether genetic factors may increase susceptibility. In this study, we explored whether certain immune system-related genetic traits (HLA types) are a potential risk factor for developing NS or other forms of onchocerciasis-associated epilepsy (OAE). In Mahenge, Tanzania, we compared human leukocyte antigen (HLA) gene variants in people with epilepsy—including NS—and healthy controls. Although we did not find statistically significant associations after rigorous correction, some HLA alleles appeared linked to disease before adjustment, including one (HLA-B*35:01) previously reported in South Sudan. These preliminary signals suggest that genetics could play a role in NS, but much larger collaborative studies will be needed to know for sure. Meanwhile, the best prevention remains stopping parasite transmission through effective onchocerciasis elimination programmes.

## Introduction

Nodding syndrome (NS) is a mentally and physically disabling disease that mainly affects children between the ages of 5 and 15 years old leading to progressive cognitive dysfunction, neurological deterioration, stunted growth, and a characteristic nodding of the head [1]. Initially described by Jilek-Aall in Mahenge village of Southern Tanzania in 1970 [2], NS was considered a mysterious neurological disease occurring exclusively in onchocerciasis-endemic areas in Uganda, South Sudan and Tanzania. Today, NS has been recognized, together with Nakalanga syndrome, as part of onchocerciasis-associated epilepsy (OAE) and has been reported in several onchocerciasis-endemic regions with high prevalence of epilepsy and high ongoing or past *Onchocerca volvulus* transmission, such as in the Democratic Republic of the Congo, South Sudan, Cameroon and the Central African Republic [3–5].

The pathophysiological mechanism of OAE, including NS, remains to be elucidated. Thus far, the only identified risk factor for OAE is the degree of *O. volvulus* infection, specifically the *O. volvulus* microfilarial load [6]. In onchocerciasis-endemic regions, a high prevalence of OAE has been observed among households living near breeding sites of blackflies (the vector of onchocerciasis) [7]. Many children in these households may suffer from OAE, but not all individuals with a high microfilarial load develop OAE [6]. Therefore, co-factors, such as genetic factors, may play a role in the aetiology of OAE.

The Human Leukocyte Antigens (HLA) or Human Major Histocompatibility complex (MHC) are a group of proteins encoded by HLA genes on the short arm of the sixth chromosome [8,9]. The HLA complex is one of the most gene-dense and diverse regions of the human genome and has been linked to a series of genetic and infectious diseases [10–12]. Certain HLA types, especially HLA-class II variants, have been associated with either immunity or increased susceptibility to generalized or localized onchocerciasis [13]. More specifically, a correlation was found between allelic variants of HLA-DQA1 (protective: *04:01; risk: *01:02, *01:03 and *03:01) and changes in clinical manifestations in north-western Ecuador [14]. In a case-control study including 48 patients with NS and 51 healthy controls conducted in South Sudan’s Mundri Area by Benedek *et al.*, an association was reported between NS and the HLA haplotype: HLA-B*42:01, C*17:01, DRB1*03:02, DQB1*04:02, DQA1*04:01, and the susceptible motif: Ala24, Glu63 and Phe67 in the HLA-B peptide-binding groove of HLA-B*35:01, *51:01 and *53:01 [15]. These amino acids create a hydrophobic and sterically closed peptide-binding HLA pocket, favoring the proline residue [15]. However, five cases in the South Sudanese study were from the same family, and cases and controls were from different villages and ethnicities. Moreover, cases and controls were not tested for onchocerciasis. Hence, it is possible that the observed HLA association was related to the families and ethnicities involved in the study or to susceptibility to onchocerciasis rather than NS.

While previous studies in Mahenge have focused primarily on infectious and environmental contributors, little is known about host genetic susceptibility [16–18]. To further investigate the potential association between OAE/NS and HLA haplotypes, a case-control study was conducted in 2021–2022 in Mahenge, an onchocerciasis-endemic area with a high-prevalence of OAE/NS in Tanzania [17,19].

## Materials and methods

### Ethics statement

The study was conducted according to the guidelines of the Declaration of Helsinki. Ethical clearance was obtained from the Ethics Committee of the National Institute for Medical Research, Tanzania (NIMR/HQ/R.8a/Vol.IX/3746) and the Ethics Committee of the Antwerp University Hospital, Belgium (B300201837863). Written consent was obtained from all participants. After informed consent/assent was obtained, participants were interviewed and examined by a physician to confirm the diagnosis of OAE/NS using a standardized questionnaire as described previously [26].

### Study setting

**Fig 1:**
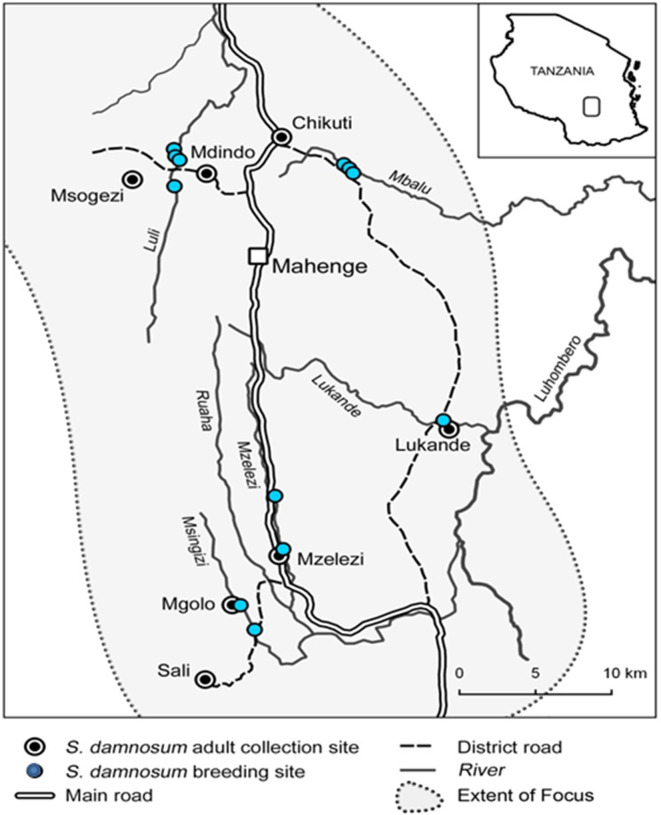
Map of Mahenge Area showing the ten surveyed villages (map prepared by A Hendy). **[**16,20**]**.

A case-control study was conducted in Mahenge, Ulanga District, Tanzania, in 2021–2022. Participants were selected from individuals previously identified with epilepsy during door-to-door surveys conducted in 2017, 2018 and 2021 [17–19]. These participants came from nine rural villages in the Mahenge area (Mzelezi, Mdindo, Sali, Msogezi, Ebuyu, Euga, Isyaga, Isongo and Mgolo) and a suburban village (Vigoi) (Fig 1).

The fast-flowing rivers in the Mahenge Mountains are known to be suitable breeding sites for blackflies [21]. The surrounding area was infamous for having a high onchocerciasis endemicity before the implementation of annual community-directed treatment with ivermectin (CDTI) in 1997 [22–24], which was switched to biannual CDTI in 2019 [19]. The Wapogoro are the predominant ethnic group in the area. These people are sustaining themselves through agriculture, livestock farming (chickens, goats and pigs, with the latter mostly kept indoors and in suburban villages) and gem mining. Other filarial worms are not known to be endemic in the area [16].

### Study design

The study was designed to be an age, sex and village-matched case-control study. However, due to widespread misinformation and distrust surrounding public health initiatives during the COVID-19 pandemic at the time of the study, it was difficult to find controls in certain villages. Many individuals refused to participate because they feared the study was conducted to vaccinate people. Therefore, when a matching control was unavailable in the village of a case, one was sought in adjacent villages.

The first phase of this study explored whether *Mansonella perstans* infection could be a risk factor for OAE [25]. Results showed that *M. perstans* was not associated with OAE in Mahenge, as none of the cases and controls tested positive for the parasite [25]. Additionally, a statistically significant association was found between anti-Ov16 seropositivity and epilepsy, OAE and probable NS. Moreover, residing longer in the village and having a family history of seizures were positively correlated with Ov16 status and made persons at higher odds for epilepsy, including NS [25]. These findings reinforced the link between onchocerciasis and epilepsy in the endemic area. Consanguine marriage within the family was reported by 8% of cases and 3.8% of controls (p = 0.26). A detailed description of the study setting, and the sociodemographic characteristics of the study population can be found in the previously published paper [25].

In this second phase, we expand the investigation by analyzing HLA haplotypes as a potential genetic risk factor for NS. We examined a subset of cases and controls with collected dried blood spots for HLA testing, aiming to determine whether specific haplotypes increase vulnerability to OAE and by extension NS.

### Study participants

A case of epilepsy was defined according to the International League Against Epilepsy (ILAE) as an individual with a history of at least two unprovoked seizures occurring 24 hours or more apart [27]. Epilepsy cases and controls without epilepsy were identified based on their positive or negative screening results for epilepsy in the previous door-to-door surveys [17–19]. A case of probable NS was defined according to the 2013 modified consensus case definition of NS [21]. A person with epilepsy was considered to be a case of OAE if the following criteria were met: 1) epilepsy onset between the ages of three and 18 years; 2) without an obvious cause of epilepsy, and; 3) living for at least three years in the study area, known to be endemic for onchocerciasis and to have geographic and familial clustering of epilepsy cases [28].

### Laboratory procedures

Dried blood spots (DBS) were collected from all participants after a single finger prick and air-dried for further storage. The DBS were tested in the laboratory with the commercially available Ov16 IgG4 rapid diagnostic test (Ov16RDT) kit (Standard Diagnostics Gyeonggi-do, Republic of Korea) following manufacturer instructions.

### HLA haplotyping

DNA was extracted from DBS, and HLA sequencing-based typing was performed by Histogenetics (Ossining, NY, USA). A total of 11 HLA loci (HLA-A, -B, -C, -DRB1, -DRB3, -DRB4, -DRB5, -DQA1, -DQB1, -DPA1, and -DPB1) were exon sequenced. Exons 2 and 3 were sequenced for HLA class I, and exon 2 for HLA class II. This method only sequences the protein-coding regions of target genes.

### Bioinformatic analysis

The resulting HLA-typed dataset (available in S1 File) was analysed using pyHLA (v4) [29], incorporating Bonferroni as a multi-test adjustment, to test for associations with *O. volvulus* anti-Ov16 seropositivity, epilepsy, OAE and NS. PyHLA [29] is a command line tool designed to test for associations between HLA alleles and disease. All association analyses were performed separately to identify HLA association nuances that might impact the clinical characteristics of the disease. Each NS case was defined as an OAE case, and each OAE case as an epilepsy case. Anti-Ov16 seropositivity as a proxy for onchocerciasis infection was analysed separately as a potential confounder for HLA associations. Due to the small sample size of our case-control study, we also considered the non-adjusted p-values to identify alleles potentially linked to disease to be investigated further in larger datasets. Additional data exploration was performed using R and RStudio, and confidence intervals were calculated following the Wald method using the prop.test() command.

Post-hoc power analyses were calculated per allele using R packages « pwr », to calculate the sample size needed to achieve the necessary power, and « epiR » to calculate the achieved power with the current sample size (n = 210), with hypnotised effect size (OR) of 2, alpha defined as 0.05, and power set at 0.95.

Genetic population statistics were calculated using “pypop” (v1.2.1), performing the Guo and Thompson test for Hardy Weinberg equilibrium (HWE), Slatkin’s implementation of the Ewens-Watterson (EW) homozygosity test of neutrality, and multi-locus pairwise linkage disequilibrium (LD) estimations.

## Results

**Fig 2:**
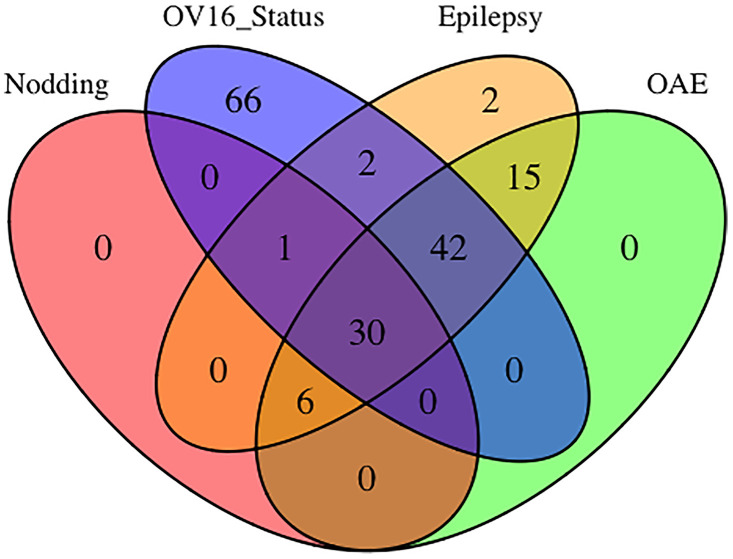
Venn diagram of population distribution based on epilepsy, onchocerciasis-associated epilepsy, nodding syndrome and anti-Ov16 seropositivity.

A total of 98 persons with epilepsy and 112 controls were included in the HLA study (Table 1). There were no statisticaly significant differences in socio-demographic characteristics of persons with epilepsy and controls for sex (p-value (p)=0.86), age (p = 0.93) and ethnicity (p = 0.10). Most persons with epilepsy (cases) met the criteria of OAE (93, 94.9%) and about one-third (37, 37.8%) met the criteria of probably NS (Fig 2). Only one person with NS (male; 45 years old) was identified with nodding syndrome with seizure onset at age five did not meet the strict OAE criteria because it was not clear whether there were other causes for epilepsy during childhood. Cases had a significantly higher prevalence of onchocerciasis-related skin disease than controls (4.1% versus 0.0%, p = 0.05), and a non-significantly higher prevalence of onchocercal nodules (19.4% versus 10.7%, p = 0.08). Anti-Ov16 seroprevalence was significantly higher among cases (76.5%) than controls (58.9%, p = 0.01). Most cases and controls (>90%) had taken ivermectin in the past.

**Table 1:** Demographic and clinical characteristics of the study population.

Study population characteristics	Persons with epilepsy (n = 98)	Controls (n = 112)	p-value^a^
**Sex** Male/Female (% of males)	46/52 (46.9%)	55/57 (49.1%)	0.86
**Age** Median (min-max) years	28 (7-63)	27 (6-65)	0.93
**Ethnicity** Hehe/Mndamba/Mpogolo	4/0/94	1/1/110	0.10
**Onchocerciasis-associated epilepsy** n/T (%; 95%CI)	93/98 (94.9%; 87.9-98.1%)	–	–
**Nodding** n/T (%; 95%CI)	37/98 (37.8%; 28.3-48.2%)	–	–
**Onchocerciasis-related skin disease** n/T (%; 95%CI)	4/98 (4.1%; 1.3-10.7%)	0/112 (0%; 0-4.1%)	0.05
**Onchocercal nodules** n/T (%; 95%CI)	19/98 (19.4%; 12.4-28.9%)	12/112 (10.7%; 5.9-18.3%)	0.08
**Anti-Ov16 seropositivity** n/T (%; 95%CI)	75/98 (76.5%; 66.7-84.3%)	66/112 (58.9%; 49.2-68.0%)	0.01
**Ivermectin intake** n/T (%; 95%CI)	91/98 (92.9%; 85.3-96.8%)	107/112 (95.5%; 95.5-98.3%)	0.59

CI – confidence interval; n – number; T – Total

^a^Differences in sociodemographic variables between cases and controls were studied using the chi-squared test for categorical variables (or Fisher’s exact test if counts <5) and the t-test for continuous variables.

After applying the Bonferroni correction for multiple comparisons, no significant differences in HLA patterns were observed between persons with epilepsy and controls (p = 0.36). Similarly, no specific HLA patterns were associated with OAE (p = 0.26), probable NS (p = 0.29) or anti-Ov16 seropositivity (p = 0.46). However, several HLA patterns showed potential relevance before multiple test adjustment (Table 2): Considering non-adjusted p-values, HLA-C*07:01 was associated with epilepsy, HLA-DQB1*06:02 was associated with OAE, HLA-B*35:01 was associated with probable NS, and HLA-C*08:02 and HLA-DRB1*03:01 were associated with anti-Ov16 seropositivity. All frequency tables based on the different case-control datasets (epilepsy/OAE/NS/anti-Ov16 seropositivity) are available in S1 File.

**Table 2:** Association statistics of identified alleles of interests and post-hoc power analysis.

	Allele	P_non-adj_	P_adj_	OR (95%CI)	Freq.	Achieved power (n = 210)	N_needed_
**Epilepsy**	C*07:01	0.02	0.36	2.35 (1.14-4.86)	0.09	0.63	507
**OAE**	DQB1*06:02	0.03	0.26	1.67 (1.08-2.58)	0.26	0.65	244
**NS**	B*35:01	0.04	0.71	4.89 (1.19-20.00)	0.02	0.12	2145
**Anti-Ov16**	C*08:02	0.05	0.79	4.64 (1.06-20.28)	0.05	0.23	868
	DRB1*03:01	0.04	0.64	0.43 (0.20-0.93)	0.14	0.50	350

OAE – Onchocerciasis-associated epilepsy; NS – Nodding syndrome; P_non-adj_ – p-value without Bonferroni adjustment; P_adj_ – p-value with Bonferroni adjustment; OR – Odds ratio; CI – confidence interval; Freq. – frequency; N_needed_ – Sample size needed to achieve sufficient power; n – number

Based on the post-hoc power analysis the minimal sample size to provide enough power would be 219 based on the highest frequency allele, whereas the needed sample size for the lowest perceived frequency would be 16,539. With the sample size used, we reached a minimal power of 0.05 and maximally 0.70, depending on the allele frequency and effect size. To provide further context, Table 2 presents the achieved power and the estimated sample size required for each of the alleles identified.

Population statistics showed that all loci, except for DRB3, presented in HWE. However, looking at individual genotypes, HLA class I (A, B, and C) show at least 11 genotypes deviating from HWE. As for class II, incl. DRB1, DQB1, DQA1, DPB1, DRB3, DRB4, DRB5 and DPA1, showed maximally 6 genotypes deviating from HWE. From the previously identified alleles, C*07:01 was shown to deviate from HWE in co-occurrence with C*17:01 (p = 0.02), as did DQB1*06:02 + DQB1*04:02 (p = 0.02). While testing for neutrality in the population, loci B and DRB1 showed significant deviation (p = 0.01, p = 0.04 respectively) from neutrality.

## Discussion

In this case-control study conducted in Mahenge, Tanzania, no specific HLA patterns could be statistically significantly associated with epilepsy, OAE, probable NS nor to anti-Ov16 seropositivity. A case control study conducted in 2020 by Benedek *et al*. in Mundri, South Sudan, suggested that NS was linked to specific HLA patterns [15]. However, the HLA patterns described by Benedek et al were not associated with epilepsy, including OAE and NS, in our study population in Mahenge [15]. Both studies had limited sample sizes, so comparisons based on non-adjusted p-values may still provide useful insights. Benedek *et al*. also reported additional HLA alleles linked to NS with significant p-values before adjustment [15]. Similar to Benedek et al. our study also identified HLA-B*35:01 to be significantly associated with NS before adjustment. Given that both studies independently identified this specific HLA haplotype to be a potential risk factor for developing NS, and Benedek *et al.* also found a specific HLA-B binding groove susceptible motif (Ala24, Glu63, and Phe67) to be significantly present in this haplotype, HLA-B*35:01 might be an interesting focus for further investigation.

Considering non-adjusted p-values, HLA-C*07:01 was perceived to be a risk factor for epilepsy. This allele has been linked to febrile infection-related epilepsy syndrome (FIRES), a rare neuroinflammatory disease characterised by a febrile illness prior to seizures onset with high morbidity in previously healthy children and young adults [30,31]. FIRES’s pathogenesis remains unknown, with current hypotheses pointing to a cytokine-mediated autoinflammatory mechanism rooted in microglial inflammation [31].

Similarly, considering non-adjusted significant p-values, HLA-DQB1*06:02 was associated with OAE. This allele has been linked to several auto-immune diseases, such as multiple sclerosis, systemic lupus erythematosus, narcolepsy and sarcoidosis [32]. Also based on non-adjusted p-values, HLA-C*08:02 and HLA-DRB1*03:01 were associated with the presence of Ov16 antibodies. However, none of these HLA types have previously been reported in association with *O. volvulus* antibody seropositivity, which has mostly been linked to HLA-DQ alleles [13,14]. However, HLA-DRB1*03:01 has been reported as a major risk factor for systemic lupus erythematosus [33] and acts as a disease modifier for autoimmune hepatitis type 1 [34].

Population statistics indicated that DRB3 was not in HWE, suggesting other driving factors of HLA diversity at this locus. These factors might be due to biological reasons such as selection pressure, population structure, or non-random mating [35]. As the ethnic groups are generally well matched, the Wahlund effect on this locus should be minor. Moreover, familial ties were kept to a minimum during case-control selection (8%), thereby minimal impact of non-random mating due to consanguinity is to be expected. Selection pressure could underlie this disequilibrium but no DRB3 alleles were linked to epilepsy in this study. However, due to the nature of the environment, multiple other infectious diseases are common in the population and could therefore drive this selection. This is in line with the well-documented tendency of deviation from HWE in HLA that are not uncommon as the immune-profile of populations are primarily driven by the pathogens circulating in the environment [36]. Nevertheless, the small sample size may also contribute to the observed equilibrium, which also extents to the deviation found in the individual alleles. Notably, two of our previously identified interesting alleles, C*07:01 + C*17:01 (p = 0.002) and DQB1*06:02 + DQB1*04:02 (p = 0.017), were found to be out of HWE during multi-locus analysis. Their respective links to epilepsy and OAE warrant further investigation into the co-occurrence with these alleles. However, due to their proximity, linkage is to be expected.

Similar evolutionary forces are likely to explain the absence of neutrality in HLA-B and DRB1, where long-term pathogen-mediated balancing selection influences allele frequencies over time, complementing the short-term genotype frequency deviations seen in HWE tests.

A slightly significantly higher proportion of cases presented with skin manifestations and nodules suggesting *O. volvulus* infection. There was also a significant difference in anti-Ov16 seropositivity between cases and controls. This is in line with the previously reported larger matched case-control study in which anti-Ov16 seroprevalence was positively associated with probable NS (OR: 5.05, 95%CI: 1.79-14.27) and overall epilepsy (OR: 2.03, 95%CI: 1.07-3.86) [25].

The pathogenesis of OAE, including NS, remains unresolved [37]. Epidemiological data indicate that higher microfilarial loads in childhood and the typical onset age (≈8–12 years) are important correlates of risk [37–39]. Cross-reactive autoimmunity (molecular mimicry with host neuronal proteins) has been proposed but remains unconfirmed and contested [40–42]. Evidence of chronic neuroinflammation, including gliosis and activated microglia on neuropathology [42], and immune/complement activation, supports an inflammatory contribution [43]. Although, cerebrospinal fluid cytokine signals have been inconsistent across small studies [44]. Macrophage migration inhibitory factor (MIF) may play a dual role: high-expression MIF genotypes appear protective, yet plasma MIF is elevated in NS and could amplify neuroinflammation [45]. Finally, metagenomics work has identified a rhabdovirus (OVRV1) in the *O. volvulus* virome across life-cycle stages and inducing an immunological response in people living in onchocerciasis endemic areas [46]. Studies are currently underway to test whether this virus could contribute to OAE pathogenesis.

Our case–control study confirms the now overwhelming epidemiological evidence for the association between onchocerciasis and epilepsy. As with many infectious disease-related conditions, genetic factors likely play a role; however, our study did not identify a clear association with any specific HLA haplotype. NS is certainly not a genetic disease. The primary risk factor for NS remains exposure to *O. volvulus*. It is crucial that both affected communities and public health decision-makers understand this, as NS is a preventable condition. Strengthening onchocerciasis elimination programs—such as switching from annual to bi-annual CDTI as implemented in Mahenge—dramatically reduced the incidence of NS [47]. Elimination of onchocerciasis transmission was shown in Uganda to also eliminated NS [48].

One major limitation of this study is the limited sample size. HLA genes are predominantly linked to ethnicity and are high in polymorphisms, making the ideal sample size for accurately representing a population and its disease associations exceptionally large. In addition, we were unable to compare our findings with HLA data from other African populations, which is particularly important given the extensive variation in HLA genes across Africa. However, due to the high costs of HLA typing, we were only able to conduct a case-control study with a limited number of participants. Based on the post-hoc analysis, expanding HLA typing of samples collected in future case-control studies and combining datasets from across sub-Saharan Africa will be needed to reach a sufficient sample size to identify potential genetic risk factors for OAE, including NS.

## Conclusion

In our case-control study in Mahenge, Tanzania, no HLA patterns were found to be statistically significantly associated with epilepsy, OAE, probable NS or anti-Ov16 seropositivity. However, several alleles—particularly HLA-B*35:01, also reported in a previous South Sudanese study [15]—emerged as potential candidates for further investigation. A meta-analysis combining our data with that from the study conducted in South Sudan should be considered. Ultimately, larger case-control studies across sub-Saharan Africa will be essential to validate these potential associations and better understand the potential genetic factors contributing to OAE and NS.

## Supporting information

S1 File Study data. (XLSX)
